# Beyond Seek and Destroy: how to Generate Allelic Series Using Genome Editing Tools

**DOI:** 10.1186/s12284-020-0366-y

**Published:** 2020-01-28

**Authors:** Leo Herbert, Anne-Cécile Meunier, Martine Bes, Aurore Vernet, Murielle Portefaix, Franz Durandet, Remy Michel, Christian Chaine, Patrice This, Emmanuel Guiderdoni, Christophe Périn

**Affiliations:** 1 CIRAD, UMR-AGAP, F-34398 Montpellier, France; 2 Université de Montpellier, Cirad, Inra, Montpellier SupAgro, F-34000 Montpellier, France

## Abstract

Genome editing tools have greatly facilitated the functional analysis of genes of interest by targeted mutagenesis. Many usable genome editing tools, including different site-specific nucleases and editor databases that allow single-nucleotide polymorphisms (SNPs) to be introduced at a given site, are now available. These tools can be used to generate high allelic diversity at a given locus to facilitate gene function studies, including examining the role of a specific protein domain or a single amino acid. We compared the effects, efficiencies and mutation types generated by our LbCPF1, SpCAS9 and base editor (BECAS9) constructs for the *OsCAO1* gene. SpCAS9 and LbCPF1 have similar efficiencies in generating mutations but differ in the types of mutations induced, with the majority of changes being single-nucleotide insertions and short deletions for SpCAS9 and LbCPF1, respectively. The proportions of heterozygotes also differed, representing a majority in our LbCPF1, while with SpCAS9, we obtained a large number of biallelic mutants. Finally, we demonstrated that it is possible to specifically introduce stop codons using the BECAS9 with an acceptable efficiency of approximately 20%. Based on these results, a rational choice among these three alternatives may be made depending on the type of mutation that one wishes to introduce, the three systems being complementary. SpCAS9 remains the best choice to generate KO mutations in primary transformants, while if the desired gene mutation interferes with regeneration or viability, the use of our LbCPF1 construction will be preferred, because it produces mainly heterozygotes. LbCPF1 has been described in other studies as being as effective as SpCAS9 in generating homozygous and biallelic mutations. It will remain to be clarified in the future, whether the different LbCFP1 constructions have different efficiencies and determine the origin of these differences. Finally, if one wishes to specifically introduce stop codons, BECAS9 is a viable and efficient alternative, although it has a lower efficiency than SpCAS9 and LbCPF1 for creating KO mutations.

## Introduction

The demonstration in 2012 and 2013 of the use of the site-specific nuclease CAS9 in eukaryotic systems to precisely mutagenize a DNA region was the first milestone in a revolution in functional biology (Cong et al. 2013; Jinek et al. 2012; Mali et al. 2013). Now commonly used in functional analysis, this technology has considerable potential for molecular breeding based on the impact of variant single nucleotides on traits of agronomic interest (Es et al. 2019). It is now possible to evaluate and reproduce the effect of a molecular polymorphism in plants and to test its effects on one or more agronomic traits (see, for instance, (Rodriguez-Leal et al. 2017; Zhou et al. 2019; Zsogon et al. 2018)).

CAS9 is an endonuclease guided by an RNA and is currently the most widely used site-specific nuclease. The first-generation ‘base editor’ was later developed to introduce specific base changes in a target sequence. This was first accomplished by creating chimeric proteins merging a CAS9 nickase (nCas9), in which either the HNH or the RuvC-like catalytic site has been inactivated, with a cytidine deaminase (Komor et al. 2016; Lu and Zhu 2017). These base editors allow targeted mutations to be induced without the integration of foreign DNA and without double-strand breakage. nCAS9 can always attach itself to a specific region and cut the nontargeted strand. The cytosine of the ssDNA is then converted to a uracil by the cytosine deaminase, and then, the uracil is replaced by a thymine during the cell cycle or by repair (Komor et al. 2016). Uracil DNA glycosylase (UDG) catalyzes the removal of U from DNA in cells and initiates base-excision repair (BER), including reversion of the U:G pair to a C:G pair. In second generation BECAS9, adding UGI (Uracil Glycosylase Inhibitor) via fusion increases the efficiency of BE by 3-fold. This technology can be used to introduce a given SNP but also to introduce a stop codon at a specific position, a technology called iSTOP (Billon et al. 2017).

CRISPR-Cpf1 (CAS12a) is a new site-specific nuclease that differs significantly from SpCAS9 (Zetsche et al. 2015). The protospacer used is T rich, i.e., ‘TTTN’, compared to that of SpCAS9, which is GC rich, i.e., ‘NGG’, allowing it to target regions rich in AT nucleotides, and it creates nucleotide overhangs while SpCAS9 creates blunt double-strand breaks. The mutations induced after repair by these two site-specific nucleases are different, potentially generating distinct alleles.

This set of gene editing tools enables the generation of a greater diversity of mutations that will facilitate the mutational and functional dissection of a given gene. Molecular diversity is a key element in generating phenotypic diversity, which can allow access to the function of a gene or be used in selection. For example, CRISPR/CAS9 technology has been used to generate a series of alleles in the promoter of a gene involved in fruit size in tomato (Rodriguez-Leal et al. 2017), redomesticate wild crop relatives (Lemmon et al. 2018) and improve agronomic traits in rice (Zhou et al. 2019).

The development and comparison of genome editing systems frequently uses marker genes to phenotypically identify visually introduced mutations. Phytoene desaturase (PDS), therefore inactivation, leads to an Albino phenotype (Qin et al. 2007). These two systems have been used frequently to test the effectiveness of the CRISPR/CAS9 system or HR (Homologous Repair) replacement rates (see (Charrier et al. 2019; Wilson et al. 2019) for two recent examples). Nevertheless, there are several limitations to their use. The mutation in the PDS gene is lethal, making it difficult to analyze molecular lesions and, above all, to transmit these lesions to subsequent generations.. When the *CAO1* gene is mutated, the plants are shorter, with a pale-yellow phenotype, but remain fertile (Miao et al. 2013). The mutation is semi-dominant and fertile and therefore makes it possible to follow the mutations over several generations. The *CAO1* gene therefore seems to be a good model for estimating the type of mutations and effectiveness of different GE systems and testing the transmission of mutations over generations. We propose, and illustrate in this paper, the value of using the *CAO1* gene as a marker gene to test the efficacy and type of mutations generated by GE and propose also that it can be used in the future as a complementary marker gene to the PDS in plants.

Few examples are available of comparing the efficacy and value of using SpCAS9, LbCPF1 and BECAS9 (see (Lee et al. 2019) for an example in maize) to create an allelic series for a given gene in a single experimental system. To illustrate the characteristics and complementarity of these three tools, we have chosen to mutagenize the *OsCAO1* gene (*Chlorophyll A Oxygenase 1*) (Miao et al. 2013) in rice. We also used a strategy called iSTOP to offer an alternative to the knockout (KO) of a single gene using BECAS9 (Billon et al. 2017).

## Results

### Constructs and targets

**Fig. 1:**
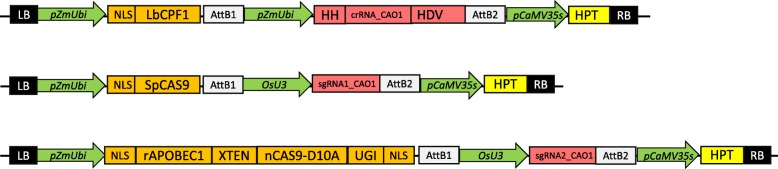
Binary plasmids used. From top to bottom. Binary plasmid containing the LbCPF1 sequence (Tang et al. 2017) codon-optimized for rice, under the control of the promoter pZmUBI; crRNA-CAO1 was also under the control of a pZmUBI promoter. The plant resistance marker was hygromycin. Binary plasmid containing SpCAS9 (Miao et al. 2013) codon-optimized for rice under the control of the pZmUBI promoter. sgRNA-CAO1 was under the control of the rice promoter pOsU3. The plant resistance marker was hygromycin. Plasmid containing BEnCAS9 (Zong et al. 2017) codon-optimized for rice (available on Addgene: #98163), formed by a fusion of the rat rAPOBEC1 protein (Komor et al.), the XTEN linker, the nCAS9 nickase having a mutation inactivating the catalytic domain RuvC (D10A) and the UGI protein. The original cloning sites were replaced by AttR Gateway recombination sites. sgRNA-BECAO1 targeting exon 3 of the *OsCAO1* gene was under the control of the rice pOsU3 promoter. HDV: HDV ribozyme; HH: Hammerhead ribozyme. All spacers were first cloned into entry vectors and then transferred to the binary vectors by LR reactions

Three binary constructs were prepared (Fig. 1). The first was designed for LbCPF1 expression, the second for SpCAS9 expression, and the last to express a nickase CAS9 (mutation D10A) fused to a rAPOBEC1 protein and a UGI protein (Tang et al. 2017). All coding sequences were placed under the control of the same promoter, pZmUBI, and flanked with one or two NLS sequences to ensure nuclear transport (Fig. 1). Finally, the selectable marker gene *HPT*, which provided rice cells with tolerance to the antibiotic hygromycin, was included in all the T-DNA constructs to facilitate comparisons between the three systems in plants.

**Fig. 2:**
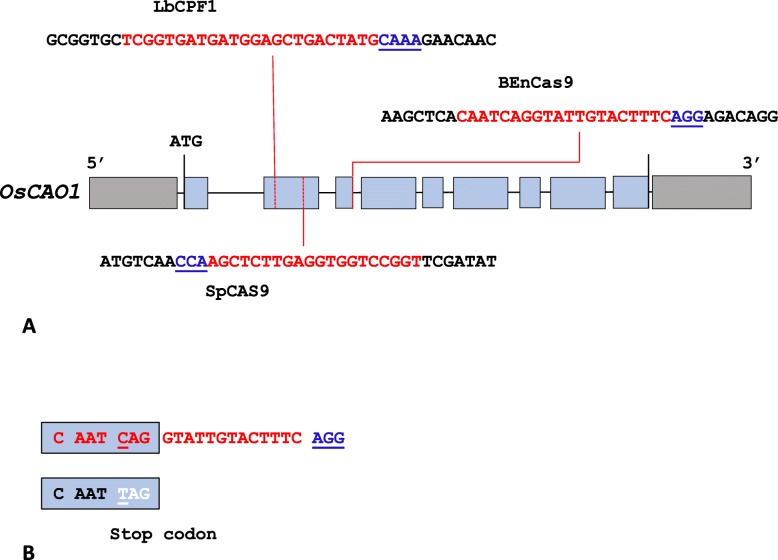
Positions and sequences of the spacers used. A) Positions of spacers and associated sequences relative to the OsCAO1 gene. PAM (CAAA (TTTN PAM on complementary strand) for LbCPF1, CCA for CAS9 (TGG (NGG on complementary strand)) and AGG for BECAS9) are shown in blue, spacers are in red, and genomic sequences are in black. B) Theoretical mutation introduced by a C- > T transversion by BECAS9, which leads to the replacement of the C nucleotide of the CAG splicing site by a T nucleotide and introduces a stop codon (TAG) instead of the amino acid glutamine

Three different spacers were designed for SpCAS9, BECAS9 and LbCPF1 targeting *OsCAO1*. *OsCAO1* exon 2 was targeted by a spacer for SpCAS9 (PAM TGG) and by a crRNA for LbCPF1 (PAM TTTG) (Fig. 2a). Exon 3 was targeted by a spacer (PAM AGG) for BECAS9 (Fig. 2a) to introduce a C- > T transversion at the CAG splicing site that converted it into a TAG stop codon. This strategy, called iSTOP, was designed to introduce a stop codon at a specific position while maintaining the reading frame (Fig. 2b).

### Mutations generated by SpCAS9, LbCPF1 and BECAS9

All three constructs were transformed into the Kitaake genotype, and 35, 25 and 44 T0 plants were generated, respectively; the mutations of these T0 lines were analyzed by PCR sequencing (Additional file 1: Table S1).

The efficiencies of the three systems, calculated as the ratio of the number of mutations observed to the number of plants regenerated and analyzed, were 94%, 72% and 36%. The SpCAS9 system was the most effective, followed by LbCPF1. In comparison, BECAS9 generated only a 36% mutation rate.

**Fig. 3:**
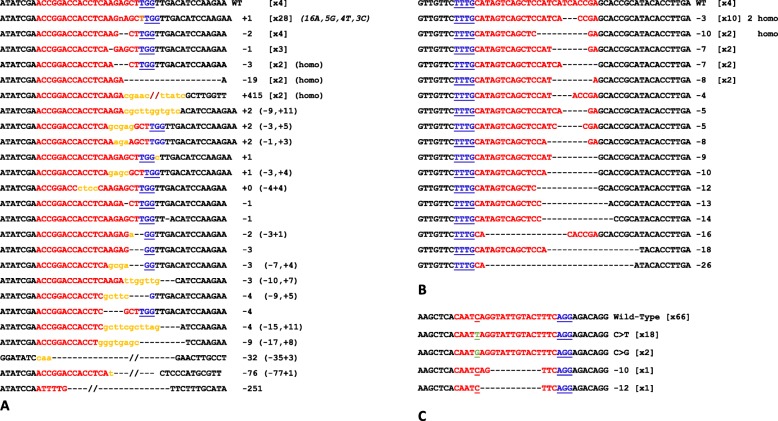
Mutations and associated frequencies generated by SpCAS9 A), LbCPF1 B) and BECAS9 C). PAMs are shown in blue, spacers in red, genomic sequences in black, inserted nucleotides in orange, and substituted nucleotides in green; − corresponds to a nucleotide deletion. For example, (− 15,+ 11) indicates a mutation due to a deletion of 15 nucleotides and an insertion of 11 nucleotides. (× 4) indicates the number of times this allele is found

The mutations were aligned with the wild-type sequences to compare the types of mutation introduced by each construct (Fig. 3). SpCAS9 mainly generated single-nucleotide insertions 3 nucleotides upstream of the PAM with a preference for the insertion of the nucleotide A (16, 5, 4 and 3 with nucleotides A, G, T and C, respectively) (Fig. 3a). The PAM was also deleted in 36% of the mutants analyzed. For LbCPF1, the situation was completely different. We did not observe any insertions but only deletions of variable size, ranging from 3 to 26 nucleotides, downstream of the PAM (100% of the mutants analyzed). Moreover, in all plants analyzed, the PAM was always present, unlike in SpCAS9 (Fig. 3b). Finally, BECAS9 generated, as expected, mostly C- > T transversions located 16 nucleotides upstream of the PAM (82% of the mutants analyzed). More surprisingly, we also identified C- > G transversions (10% of the mutants analyzed) and deletions of − 10 and − 12 nucleotides (20%) located 3 nucleotides upstream of the PAM (Fig. 3c).

### Comparison of the three systems for generation of homozygous, heterozygous and biallelic mutations

SpCAS9 was the nuclease that generated the most homozygous mutations (37%) compared to LbCPF1 and BECAS9 (12% and 14%) (Table 1). LbCPF1 and SpCAS9 produced similar proportions of biallelic mutants (46% and 40%). The three constructs induced comparable rates of heterozygosity, between 11 and 18%, and the inefficiency of each of the constructs, measured by the number of wild-type plants produced, was on the order of 6% for SpCAS9, 28% for LbCPF1 and 63% for BECAS9 (Table 1).

**Table 1:** Homozygous, heterozygous, biallelic and wild-type plants produced using SpCAS9, LbCPF1 and BE_nCAS9D10A expressed as % of analyzed regenerated plants

	Homozygous	Biallelic	Heterozygous	Wild type
SpCAS9 (*n* = 35)	37.1% (13)	45.7% (16)	11.4% (4)	5.7% (2)
LbCPF1 (*n* = 25)	12% (3)	40% (10)	16% (4)	28% (7)
BE_nCAS9D10A (*n* = 44)	13.6% (6)	0% (0)	22.7% (10)	63.6% (28)

If we first analyze the ability to restore the open reading frame, significant differences appear between SpCAS9 and LbCPF1 (Table 2). LbCPF1 generated 4 times more in frame deletion than SpCAS9. SpCAS9 mutations are mostly (90%, Table 2) out frame mutations. LbCPF1 produced only simple deletions without insertions (100%, Table 3) while the vast majority of SpCAS9 mutations are insertions (52%) and complex insertion/deletions (21%). Although BECAS9 generated mainly C- > T transversions, we also found small deletions in 2% of the mutants analyzed (Table 3).

**Table 2:** Deletions out of frame (Out-frame), deletions that maintain reading frame (In-Frame) generated using SpCAS9, LbCPF1 and BE_nCAS9D10A expressed as % of total mutant alleles identified. A total of 60, 30 and 22 mutants alleles were identified for SpCAS9, LbCPF1 and BE_nCAS9D10A respectively (see also Fig. 3)

	InFrame	OutFrame
SpCAS9	10% (6)	90% (56)
LbCPF1	43% (13)	57% (17)
BE_CAS9	95% (21)	5% (1)

**Table 3:** Deletions, insertions, deletions plus insertions (Ind/del) and substitutions generated using SpCAS9, LbCPF1 and BE_nCAS9D10A expressed as % of total mutant alleles identified. A total of 60, 30 and 22 mutants alleles were identified for SpCAS9, LbCPF1 and BE_nCAS9D10A respectively (see also Fig. 3)

	Deletions	Insertions	Ind/del	Substitutions
SpCAS9	27% (16)	52% (31)	21% (13)	0% (0)
LbCPF1	100% (30)	0% (0)	0% (0)	0% (0)
BE_CAS9	9% (2)	0% (0)	0% (0)	91% (20)

**Fig. 4:**
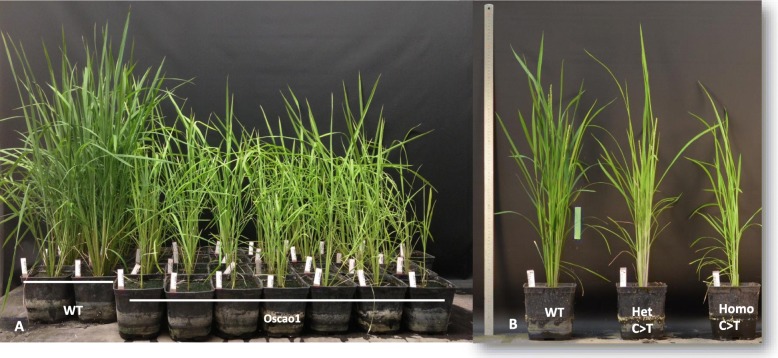
Phenotypes associated with mutations generated by CAS9 (A) and BECAS9 (B). WT = wild type, Oscao1 = mutant induced by CAS9. (A) Twenty-nine mutants are visible among 33 mutant plants: yellow plants smaller than WT. (B) From left to right, a WT plant, a heterozygous C- > T plant, and a homozygous C- > T plant. The heterozygous and homozygous plants have a pale-yellow phenotype, as expected, and the homozygous plant is shorter than the heterozygous plant and the WT

SpCAS9 generated mainly KO mutations via frameshift leading to the appearance of a premature stop codon (PSC), and we observed the expected phenotype in most of the mutant plants (Fig. 4). In this example, when 35 mutants were compared to the control, 33 plants had mutations, and 29 had the expected pale-yellow phenotype (Fig. 4a). The two plants with phenotypes similar to WT harbored nucleotide deletions in multiples of 3 that maintained the reading frame of *OsCAO1*. Similarly, half of the LbCPF1 plants did not have a visible phenotype, because 13 of the 30 alleles contained triplet nucleotide deletions. BECAS9 generated stop codons, and a comparison of heterozygous plants for C- > T transversions allowed us to highlight a difference between the phenotype of the heterozygous and the homozygous T0 plants (Fig. 4b). Both the heterozygous and homozygous mutations yielded the yellow leaf blade phenotype, but the plants harboring homozygous mutations exhibited a more severe phenotype with a shorter habit.

## Discussion

**Mutations produced by cellular repairs induced by double-strand breaks generated by SpCAS9 are not random.** The vast majority of the mutations introduced correspond to insertions of a single base (see, for instance (Lee et al. 2019), in maize) and, to a lesser extent, to short deletions generated by cut site microhomologies (Allen et al. 2018). Moreover, these insertions of a single base are not random because of the creation of 5 nt overhangs by SpCAS9, which are filled by polymerases and then religated (Lemos et al. 2018) so CAS9 induced in majority duplications of the fourth nucleotide in 5′ of the PAM. Consequently, in our case, as predicted, the most frequent mutation observed corresponds to a duplication of the 4th nucleotide (A) upstream of the PAM (Lemos et al. 2018); this case was found in 16 of 68 cases, although we also detected single-nucleotide insertions of C, T, and G at lower frequencies (see Fig. 3a). It is therefore possible to predict to some extent the type of mutations introduced using a given guide for SpCAS9, although the full diversity of the mutations generated by SpCAS9 is sometimes difficult to predict and may include deletions, deletions and insertions, and insertions of other single nucleotides. SpCAS9 also frequently generates homozygous and biallelic mutations, which have a very high probability of generating KO mutations in the primary transformants, providing a rapid way to assess the function of a gene directly after transformation. However, this feature can be problematic for genes involved with regeneration, viability or fertility. GC composition affects the effectiveness of SpCAS9. It appears that the GC percentage of the area targeted by the sgRNA is correlated with the effectiveness of the SpCAS9 (Ma et al. 2015; Raitskin et al. 2019). Considering that most genes have higher GC contents in their 5′ regions this suggest the SpCAS9 is more efficient to target 5′ end genes.

**LbCPF1 mainly generates heterozygous deletions.** The cuts made by LbCPF1 have nucleotide overhangs, while the SpCAS9 cuts are blunt double-strand breaks. The overhangs are repaired more frequently by resection of the extremities and by MMEJ, inducing small deletions, which are a common result found when using LbCPF1 (Kim et al. 2016; Lee et al. 2019; Li et al. 2018; Tang et al. 2017; Xu et al. 2017). These small deletions sometimes lead to a significant frequency of in-frame deletions, allowing one or more nucleotides to be eliminated while maintaining the reading frame. This feature could theoretically allow us to study the effects of one or more amino acid deletions on protein function and represents a comparative advantage of LbCPF1 over SpCAS9. Generating mainly small deletions proved also an advantage for abolishing the recognition of promoter sequences by TALE effectors of bacterial blight thereby conferring partial or complete resistance to different strains (Oliva et al. 2019).

The high number of heterozygotes generated by LbCPF1, previously reported in the literature (Li et al. 2018; Xu et al. 2017), makes it possible to study the function of genes for which KO is lethal or interferes with regeneration, while SpCAS9 mainly generates homozygous or biallelic mutants. Some LbCPF1 constructs appear to be as effective as SpCAS9 in rice and more effective than the construct we used (Tang et al. 2017). The reason is not clear and may depend on the guide used. This question would merit further comparative analysis of the different types of LbCPF1 constructs used in different publications.

**BECAS9 has proven its effectiveness as part of the iSTOP strategy and is now a mature technology despite its limitations**, particularly the C- > T conversion rate, which is variable depending on the targeted region. For example, we obtained 55% conversion of the *OsNRT1.1B-C* in *OsNRT1.1B-T* alleles (data not shown), compared to a 22% conversion rate in *OsCAO1* (our results), and the average rates obtained by (Zong et al. 2017) were also approximately 43%. We also obtained significant C- > G conversion rates of approximately 10% of the mutant lines, as found by (Lu and Zhu 2017), suggesting that the system is not perfect and generates a small number of C- > G conversions that can also be used to create new alleles. Finally, as in (Lu and Zhu 2017), we found a low frequency of small deletions (10%) probably due to nickase CAS9 (D10A) activity, which nicks the unedited strand. Cytosine base editors, BECAS9 in our paper, induce genome-wide mutations in rice (Jin et al. 2019) and in mouse embryos (Zuo et al. 2019), even with a high-fidelity version of the cytosine base editor (Jin et al. 2019), and the off-targets cannot be predicted in silico. This is not the case for the adenosine base editor (Jin et al. 2019; Zuo et al. 2019), and overall, these results suggest that using the adenine base editor (Yan et al. 2018) instead of cytosine base editor in the future may be more efficient, or alternatively, that the cytosine base editor should be optimized. Moreover, the inherent constraint of BECAS9, i.e., the C- > T transversion, is mostly restricted to the − 4 to − 8 interval upstream of the PAM, limiting the accessible targets of the BECAS9 and the iSTOP strategy. Using a new BECAS9 with a new PAM and adenosine deaminase should help in the future to increase the usefulness of this approach (Hua et al. 2019).

## Conclusion

The revolution in genome editing has provided the recent possibility to generate allelic series in any gene in plants, combined with an expansion of PAM specificity that will facilitate subtle and powerful functional analysis of any gene and any protein domain. For instance, allelic series were used to analyze the function of *OsIAA23* when simple KO lines did not have any visible phenotype (Jiang et al. 2019). Removing 13 amino acids, however, induced a severe phenotype. In this example, mosaic transcripts of *OsIAA23* were produced in frameshift mutants that did not represent “true” KOs (Jiang et al. 2019). A base editor could have been efficiently used to generate predictable KOs with premature termination of transcription. Alternatively, LbCPF1 could have been employed to generate longer deletion mutations. SpCAS9, LbCPF1 and BECAS9 have complementary characteristics; for instance, LbCPF1 can be used to delete one or several amino acids in frame, while BECAS9 can be used to introduce a frameshift mutation, to modify the phosphorylation status of a single amino acid, or to introduce a stop codon or any single amino acid change. With the development of CRISPR-CAS9 tools recognizing different PAMs (Hua et al. 2019), the range of nucleotides accessible to BECAS9 will also expand, giving rise to a large set of genome editing tools for deciphering gene functions in detail. Although off-target activity of SpCAS9 has been described in most eukaryotes and is highly variable depending on the sgRNA designed (O'Geen et al. 2015; Tsai and Joung 2016), it seems that off-target mutations are rare in plants and can almost always be predicted in silico (Li et al. 2019; Tang et al. 2018; Young et al. 2019), even if there is still debate regarding the level of off-target activity in plants (Zhang et al. 2018). Far fewer data are available concerning the off-target activity of LbCPF1, but the situation seems similar to that of SpCAS9 (Tang et al. 2018). Several complementary strategies have been developed, including the construction of high-fidelity SpCAS9, specialized deep learning software to help users reduce the probability of designing sgRNAs with high off-target potential, and technologies to predict or identify at a genome-wide level the frequencies of off-targets (O'Geen et al. 2015; Tsai and Joung 2016). Last, we think the *CAO1* gene is a marker gene complementary to the PDS gene for future study to compare GE efficiency in plants. i) it is not lethal, ii) the phenotype including heterozygotes is visible very early, iii) it is well conserved and can therefore be used in many plant species iv) It is also theoretically possible to estimate the efficacy of HDR with this gene, by restoring, for example, the framework for reading mutations in plants mutated in *CAO1*. We therefore propose its use as a marker gene to evaluate in the future the effectiveness and comparison of current and future publishing technologies.

Recently, a promising new technology derived from CRISPR/CAS9, prime-editing, has been developed. The system consists of a CAS9 nickase merged with a reverse transcriptase that is programmed by a prime editing guide (Anzalone et al. 2019). This system has the potential to introduce a very large range of variations, including deletions, insertions, base modifications and modifications combining all three at once. This system represents the next frontier of genome editing in plants and rice to be implemented.

## Material and methods

### CRISPR design

The targets in *OsCAO1* (LOC_Os10g41780) for LbCPF1, BECAS9 and SpCAS9 were defined with the CRISPOR-Tefor software (http://crispor.tefor.net/) by minimizing the number of possible off-targets (Additional file 1**: Table S1**). The crRNA for BECAS9 was designed to introduce a stop codon by transversion of a C to T in position − 4 to − 8 of the PAM, the most favorable position for the use of cytosine deaminase, after analysis of the complete gene sequence on OryGenesDB (http://orygenesdb.cirad.fr/) (Komor et al. 2016; Zong et al. 2017).

### Cloning spacers in pentry vectors

Cloning was carried out in entry vectors by digestion ligation using *Bsa* I sites. Briefly, the forward and reverse primers (Additional file 1: Table S1) corresponding to each spacer having sites complementary to the input vectors were ordered (Promega, USA) and then annealed: primers (100 μM, 2.8 μL each in 50 μL final volume) were denatured at 95 °C for 5 min and then allowed to stand at room temperature for 1 h. Each entry vector (2 μg) was digested with *Bsa* I restriction enzyme (40 U) in a 50 μL final volume. Six microliters of the annealed primers was added for the ligation step together with the plasmid DNA of each entry vector (100 ng, 2.5 μL), 10x ligase buffer (2 μL), and T4 ligase (400 UI) in a final volume of 20 μL. The ligation reaction was performed at 16 °C overnight. Then, 4 μL of each ligation product was transformed into 50 μl of chemocompetent DH5 alpha bacteria by heat shock. Positive clones were identified by *Bsa* I restriction (Additional file 1: Table S1).

### Cloning by LR in binary destination vectors

crRNA (LbCPF1) under the control of the ZmUbi promoter was flanked by the hammerhead (HH) and hepatitis delta virus (HDV) ribozyme RNAs for precise crRNA processing (Tang et al. 2017). The sgRNAs (CAS9 and BECAS9) were under the control of the OsU3 promoter. Coding cassettes containing the guide RNAs were transferred to the destination binary vectors pUbi_LbCPF1-destvect4.0, pZmUbi-OsCas9-HPT and pBE_nCAS9-HPT_AttR-ccdB and transformed into chemocompetent DH5 alpha bacteria by heat shock. After verification by sequencing, the plasmids were transferred by electroporation to *Agrobacterium tumefaciens* strain EHA105. A final check by restriction was carried out on plasmids extracted from *A. tumefaciens* before genetic transformation.

### Agrobacterium-mediated rice transformation

Genetic transformation was carried out in the Kitaake variety according to the protocol published elsewhere using mature seed embryo-derived secondary calluses (Sallaud et al. 2004). The selection was performed on a hygromycin medium.

### Analysis of mutations

A total of 35 SpCAS9, 25 LbCPF1 and 44 BE_nCAS9D10A plants were regenerated and analyzed by PCR sequencing using primers to amplify the areas targeted by LbCPF1, SpCAS9 and BECAS9 (Additional file 1: Table S1). The sequences obtained after cleaning were aligned with the reference sequence for each targeted locus. Mutations were analyzed manually with reference to the wild-type sequence.

## Supplementary information

**Additional file 1: Table S1.** Primers used for cloning, mutation detection and sequencing

## Data Availability

All the datasets, including videos and photographs, are included in the article and are also available from the corresponding author upon reasonable request. All the transgenic lines are also available through MTA from the corresponding author.
